# Altered gene expression in highly purified enterocytes from patients with active coeliac disease

**DOI:** 10.1186/1471-2164-9-377

**Published:** 2008-08-08

**Authors:** Suzanne Bracken, Greg Byrne, Jacinta Kelly, John Jackson, Conleth Feighery

**Affiliations:** 1 Department of Immunology, St. James's Hospital, Dublin and Trinity College Dublin, Dublin Molecular Medicine Centre, Dublin, Ireland

## Abstract

**Background:**

Coeliac disease is a multifactorial inflammatory disorder of the intestine caused by ingestion of gluten in genetically susceptible individuals. Genes within the HLA-DQ locus are considered to contribute some 40% of the genetic influence on this disease. However, information on other disease causing genes is sparse. Since enterocytes are considered to play a central role in coeliac pathology, the aim of this study was to examine gene expression in a highly purified isolate of these cells taken from patients with active disease. Epithelial cells were isolated from duodenal biopsies taken from five coeliac patients with active disease and five non-coeliac control subjects. Contaminating T cells were removed by magnetic sorting. The gene expression profile of the cells was examined using microarray analysis. Validation of significantly altered genes was performed by real-time RT-PCR and immunohistochemistry.

**Results:**

Enterocyte suspensions of high purity (98–99%) were isolated from intestinal biopsies. Of the 3,800 genes investigated, 102 genes were found to have significantly altered expression between coeliac disease patients and controls (p < 0.05). Analysis of these altered genes revealed a number of biological processes that are potentially modified in active coeliac disease. These processes include events likely to contibute to coeliac pathology, such as altered cell proliferation, differentiation, survival, structure and transport.

**Conclusion:**

This study provides a profile of the molecular changes that occur in the intestinal epithelium of coeliac patients with active disease. Novel candidate genes were revealed which highlight the contribution of the epithelial cell to the pathogenesis of coeliac disease.

## Background

Coeliac disease is a permanent intolerance to dietary prolamins from wheat, barley and rye. Ingestion of these proteins in susceptible individuals gives rise to an inflammatory lesion in the small intestine characterised by crypt hyperplasia and villous atrophy [1]. While progress has been made in understanding the mechanisms by which prolamins activate the immune system, the molecular events that ultimately lead to the intestinal lesion are, as yet, ill defined.

Coeliac disease has a strong HLA association with approximately 95% of coeliac patients expressing the HLA-DQ2 molecule [2]. A large population-based study showed the disease concordance rate between monozygotic twins to be 75% [3]. This rate is considerably higher than that for other multifactorial diseases such as Crohn's disease [4] or insulin dependent diabetes mellitus [5]. However, in the study by Greco *et al*, the concordance rate for coeliac disease in HLA-matched dizygotic twins was found to be only 11% [3]. Thus, while the evidence points to a very strong HLA genetic contribution to coeliac disease, other non-HLA-linked genes must play a role.

Additional linkage studies have been performed in coeliac disease in an attempt to identify susceptibility loci other than the 6p21 HLA locus. Evidence has been found for linkage with the non-HLA loci 2q33, 5q31-33 and 19p13 [6] and candidate gene association studies within these loci have concentrated on genes known to be immunologically relevant to disease pathogenesis. Recent genome-wide association studies have identified a region harbouring IL-2 and IL-21 as a further potential genetic susceptibility region predisposing to celiac disease [7,8]. However, so far no gene has been conclusively proven to confer a risk of coeliac disease. Hence, a hypothesis-free approach to selecting genes for study, as employed here, may be useful.

Much research in coeliac disease has focused on the role of T-cells and the pro-inflammatory cytokines they produce [9-13]. It has been suggested that the direct effect of pro-inflammatory cytokines such as IFN-γ and TNF-α may contribute to the characteristic coeliac lesion [9]. Members of the metalloproteinase (MMP) family have been implicated in coeliac disease pathology. These enzymes are capable of tissue remodelling by degradation of proteins in the extracellular matrix and basement membrane. Several studies have demonstrated elevated levels of MMP expression in the coeliac lesion [14-16].

It has been proposed that dysregulated differentiation of epithelial cells in the small intestine may also play a role in the generation of the coeliac lesion. Diosdado *et al *have suggested that stem cells in the villous crypt proliferate, but do not receive the signal to differentiate leading to the development of undifferentiated, hyperplastic crypts and subsequently, villous atrophy [17]. It has recently been reported that gliadin can directly cause up-regulation of several epithelial cell surface molecules such as HLA-DR, ICAM-1 and MICA [18]. Furthermore, other studies have reported increased expression of several cytokines in the epithelium of patients with active celiac disease including IL-15, MIF, TNF-α and iNOS [19-21]. Thus, the intestinal enterocyte is emerging as a potential contributor to coeliac disease pathogenesis and must be studied further.

The purpose of this study was to examine the role of the epithelial cell in coeliac disease, employing a gene microarray based technique. This allowed for the analysis of the simultaneous expression of thousands of gene transcripts, in a hypothesis-free manner [22]. Epithelial cells were isolated from biopsies taken from coeliac patients with active disease and compared to controls, thereby examining the gluten-induced inflammatory environment of the coeliac lesion. In the study, 102 genes were found to have significantly altered expression. Further studies using RT-PCR and immunohistochemistry were used to validate altered expression of gap junction protein alpha 4 and small proline rich protein 3.

## Results

### Microarray analysis of coeliac duodenal epithelial cells

**Figure 1:**
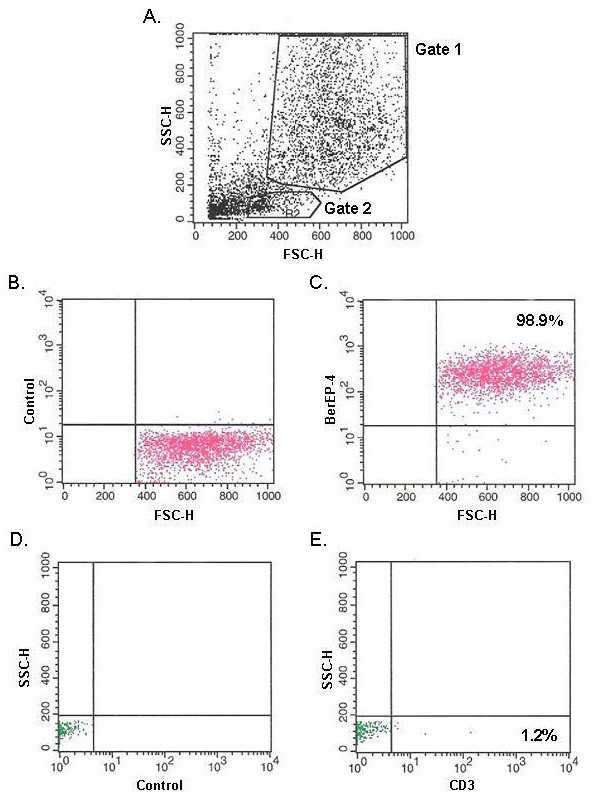
**Flow cytometric analysis of purified epithelial cells**. **A: **Size versus granularity plot. **B **and **C **correspond to the population of cells within gate 1; and demonstrate control antibody and BerEP-4 expression, respectively. **D **and **E **correspond to the population of cells within gate 2; and demonstrate control antibody and CD3 expression, respectively.

**Figure 2:**
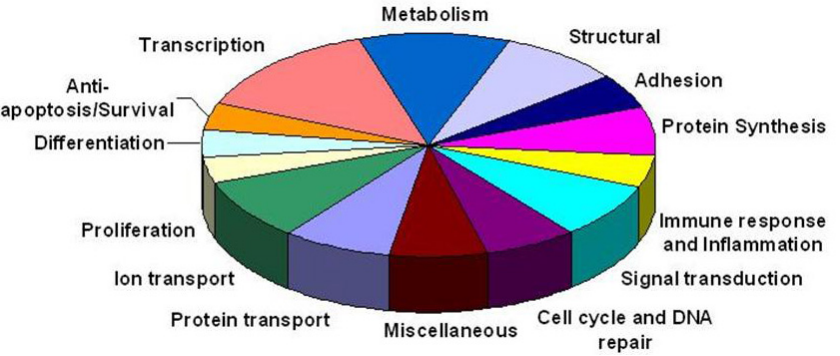
102 differentially expressed genes organised by category.

DTT/EDTA treatment was employed to strip the epithelial layer from patient intestinal biopsies. Magenetic cell sorting was then used to deplete CD3^+ ^cells, and enterocyte suspensions with purities of 98–99% were routinely obtained (Figure 1). Using Atlas Glass Human 3.8I oligonucleotide micoarrays (BD Biosciences Clontech, UK), we analysed the gene expression profile of a homogeneous population of duodenal epithelial cells taken from patients with active coeliac disease, in comparison to control patients. Of the 3,800 genes present on the array (all of which have been previously annotated), 3549 had sufficient data across the five experiments for comparison. Many of these genes showed fold-change ratios with little or no deviation from 1. Thus, to focus on only differentially expressed genes, the list was filtered on a fold-change of 1.25-fold. A fold-change of 1.25 has been described in previously published microarray experiments [23,24] and has been shown to indicate reliable differences in gene expression [23]. This filtering yielded a list of 1,256 genes with which to perform analysis. Of these 1,256 genes, 827 were up-regulated and 429 were down-regulated. A p-value of 0.05 was used as a cut-off to distinguish significantly expressed genes, which yielded a gene list of 102 (68 up-regulated and 34 down-regulated) genes which are presented in Table 1. These 102 genes were grouped according to functional categories, including protein transport, ion transport, proliferation, differentiation, anti-apoptosis/survival, structural, adhesion, metabolism, transcription, protein biosynthesis, signal transduction, cell cycle and DNA repair, and immune response and inflammation (categories of genes and up/down-regulation are summarised in Figure 2 and Table 2 respectively).

**Table 1:** Differentially expressed genes.

Functional Category	Gene Name	GenBank Accession Number	Chromosome Location	Fold-change	p-value
Protein Transport	peroxisome biogenesis factor 13	NM_002618	2p14-p16	2.276	0.00331
	gap junction protein, alpha 4, 37 kDa (connexin 37)	NM_002060	1p35.1	1.952	0.00575
	syntaxin 3A	NM_004177	11q12.1	1.81	0.00578
	CD3G antigen, gamma polypeptide (TiT3 complex)	NM_000073	11q23	1.547	0.00676
	retinol dehydrogenase 5 (11-cis and 9-cis)	NM_002905	12q13-q14	1.446	0.0158
	exportin 1 (CRM1 homolog, yeast)	NM_003400	2p15	1.847	0.0225
	A kinase (PRKA) anchor protein 1	NM_005751	7q21-q22	0.748	0.0248
	retinol binding protein 1, cellular	NM_002899	3q21-q23	0.692	0.0305

Ion Transport	gamma-aminobutyric acid (GABA) A receptor, beta 3	NM_000814	15q11.2-q12	1.74	0.00286
	cholinergic receptor, nicotinic, alpha polypeptide 5	NM_000745	15q24	0.557	0.00605
	hemochromatosis	NM_000410	6p21.3	1.319	0.00924
	potassium voltage-gated channel, Isk-related family, member 1	NM_000219	21q22.12	1.369	0.00959
	calcium channel, voltage-dependent, L type, alpha 1C subunit	NM_000719	12p13.3	1.351	0.0112
	sorcin	NM_003130	7q21.1	1.316	0.0248
	ATPase, H+ transporting, lysosomal 70 kDa, V1 subunit A	NM_001691	3q13.2-q13.31	1.47	0.0364
	glutamate receptor, ionotropic, N-methyl D-aspartate 1	NM_000832	9q34.3	0.706	0.0368
	adenosine kinase	NM_001123	10q22.2	0.749	0.0406

Proliferation	bone marrow stromal cell antigen 2	NM_004335	19p13.2	1.29	0.00975
	PRKC, apoptosis, WT1, regulator	NM_002583	12q21	0.73	0.0251
	v-Ha-ras Harvey rat sarcoma viral oncogene homolog	NM_005343	11p15.5	0.631	0.0282
	growth arrest-specific 6	NM_000820	13q34	1.576	0.0443

Differentiation	nuclear transcription factor Y, alpha	NM_002505	6p21.3	0.714	0.0196
	homeo box A7	NM_006896	7p15-p14	1.701	0.0297
	ash2 (absent, small, or homeotic)-like (Drosophila)	NM_004674	8p11.2	0.749	0.0368
	wingless-type MMTV integration site family, member 7A	NM_004625	3p25	1.33	0.044

Anti-apoptosis/Survival	PTK2B protein tyrosine kinase 2 beta	NM_004103	8p21.1	0.719	0.00362
	dual-specificity tyrosine-(Y)-phosphorylation regulated kinase 3	NM_001396	21q22.13	2.627	0.0304
	tumor necrosis factor receptor superfamily, member 18	NM_004195	1p36.3	1.941	0.0334
	Erythropoietin	NM_000799	7q22	2.738	0.0492

Transcription	SRY (sex determining region Y)-box 14	NM_006942	17p13	0.732	0.0142
	hematopoietic cell-specific Lyn substrate 1	NM_005335	3q13	1.585	0.0164
	histone deacetylase 2	NM_001527	6q21	1.574	0.0178
	high-mobility group box 3	NM_002128	13q12	1.775	0.02
	ELK4, ETS-domain protein (SRF accessory protein 1)	NM_001973	1q32	1.446	0.0216
	homeo box A11	NM_005523	7p15-p14	1.325	0.0246
	forkhead box G1B	NM_005249	14q13	1.558	0.0248
	notch homolog 3 (Drosophila)	NM_000435	19p13.2-p13.1	1.271	0.0285
	paired box gene 8	NM_003466	2q12-q14	1.419	0.0289
	human T-cell leukemia virus enhancer factor	NM_002158	2p22-p16	1.267	0.0311
	methyl CpG binding protein 2 (Rett syndrome)	NM_004992	Xq28	0.532	0.0326
	T-box 6	NM_004608	16p11.2	0.73	0.037
	TAF15 RNA polymerase II, TATA box binding protein (TBP)-associated factor, 68 kDa	NM_003487	17q11.1-q11.2	0.656	0.0432
	neuronal PAS domain protein 2	NM_002518	2q11.2	1.288	0.0497

Metabolism	fructose-1,6-bisphosphatase 1	NM_000507	9q22.3	1.535	0.000296
	glucosidase, beta; acid (includes glucosylceramidase)	NM_000157	1q21	1.258	0.000369
	3-hydroxy-3-methylglutaryl-Coenzyme A synthase 1 (soluble)	NM_002130	5p14-p13	0.535	0.00679
	lipoprotein, Lp(a)	NM_005577	6q26	1.653	0.0174
	dihydropyrimidinase	NM_001385	8q22	0.638	0.0205
	NADH dehydrogenase (ubiquinone) Fe-S protein 8, 23 kDa (NADH-coenzyme Q reductase)	NM_002496	11q13	0.78	0.0242
	histatin 1	NM_002159	4q13	0.75	0.0256
	N-acetylgalactosaminidase, alpha-	NM_000262	22q13-qter; 22q11	0.783	0.0278
	choline kinase	NM_001277	11q13.2	1.507	0.0303
	6-phosphofructo-2-kinase/fructose-2,6-biphosphatase 4	NM_004567	3p22-p21	0.648	0.032
	sterol regulatory element binding transcription factor 1	NM_004176	17p11.2	4.529	0.0354

Structural	cytoplasmic linker 2	NM_003388	7q11.23	1.309	0.00603
	procollagen-lysine, 2-oxoglutarate 5-dioxygenase (lysine hydroxylase) 2	NM_000935	3q23-q24	1.563	0.00657
	spectrin, alpha, erythrocytic 1 (elliptocytosis 2)	NM_003126	1q21	1.29	0.0108
	cystatin A (stefin A)	NM_005213	3q21	1.48	0.0114
	integrin beta 4 binding protein	NM_002212	20q12	2.356	0.0167
	envoplakin	NM_001988	17q25	0.69	0.0201
	tubulin-specific chaperone a	NM_004607	5q14.1	0.725	0.0239
	microtubule-associated protein 1A	NM_002373	15q13-qter	0.652	0.0395
	small proline-rich protein 3	NM_005416	1q21-q22	2.095	0.0398

Adhesion	vinculin	NM_003373	10q22.2	1.752	0.00217
	matrix Gla protein	NM_000900	12p13.1-p12.3	0.659	0.00909
	EGF-containing fibulin-like extracellular matrix protein 1	NM_004105	2p16	1.276	0.0273
	oligodendrocyte myelin glycoprotein	NM_002544	17q11.2	2.008	0.0386
	growth factor receptor-bound protein 7matrix Gla protein	NM_005310	17q12	1.564	0.0479

Protein Synthesis	ribosomal protein L19	NM_000981	17q11.2-q12	1.744	0.00118
	eukaryotic translation elongation factor 1 beta 2	NM_001959	2q33-q34	1.413	0.0051
	ribosomal protein S29	NM_001032	14q	1.311	0.00579
	pyrroline-5-carboxylate reductase 1	NM_006907	17q25.3	1.31	0.0059
	aminolevulinate, delta-, synthase 1	NM_000688	3p21.1	1.453	0.0105
	eukaryotic translation initiation factor 4E binding protein 3	NM_003732	5q31.3	1.466	0.0143
	tyrosinase (oculocutaneous albinism IA)	NM_000372	11q14-q21	1.272	0.0387

Immune Response & Inflammation	MAP/microtubule affinity-regulating kinase 2	NM_004954	11q12-q13	0.717	0.00307
	microseminoprotein, beta-	NM_002443	10q11.2	1.592	0.0113
	sialyltransferase 1 (beta-galactoside alpha-2,6-sialyltransferase)	NM_003032	3q27-q28	1.547	0.0207
	pyruvate kinase, muscle	NM_002654	15q22	1.438	0.031
	superkiller viralicidic activity 2-like (S. cerevisiae)	NM_006929	6p21	1.639	0.0401

Signal Transduction	natriuretic peptide receptor A/guanylate cyclase A (atrionatriuretic peptide receptor A)	NM_000906	1q21-q22	0.693	0.00936
	membrane protein, palmitoylated 3 (MAGUK p55 subfamily) member 3)	NM_001932	17q21.31	1.259	0.0109
	GPI anchored molecule like protein	NM_007264	12q13.3	1.791	0.0116
	glutamate receptor, metabotropic 7	NM_000844	3p26.1-p25.1	0.768	0.026
	G protein-coupled receptor 7	NM_005285	8p22-q21.13	2.293	0.0265
	Ras-like without CAAX 1	NM_006912	1q22	1.306	0.0356
	retinal G protein coupled receptor	NM_002921	10q23	0.744	0.0458
	AND-1 protein	NM_007086	14q22.3	1.659	0.0465

Cell cycle & DNA repair	developmentally regulated GTP binding protein 1	NM_004147	22q12.2	1.56	0.00551
	protein (peptidyl-prolyl cis/trans isomerase) NIMA-interacting, 4 (parvulin)	NM_006223	Xq13	0.628	0.0086
	flap structure-specific endonuclease 1	NM_004111	11q12	1.288	0.0167
	BRCA1 associated protein-1 (ubiquitin carboxy-terminal hydrolase)	NM_004656	3p21.31-p21.2	1.316	0.0185
	amyloid beta (A4) precursor protein-binding, family B, member 1 (Fe65)	NM_001164	11p15	2.218	0.0234
	ubiquitin protein ligase E3A (human papilloma virus E6-associated protein, Angelman syndrome)	NM_000462	15q11-q13	0.771	0.0286
	nudix (nucleoside diphosphate linked moiety X)-type motif 2	NM_001161	9p13	2.209	0.037

Miscellaneous	chromosome 18 open reading frame 1	NM_004338	18p11.2	1.743	0.0298
	prion protein (p27-30) (Creutzfeld-Jakob disease, Gerstmann-Strausler-Scheinker syndrome, fatal familial insomnia)	NM_000311	20p13	0.539	0.00875
	oxidase (cytochrome c) assembly 1-like	NM_005015	14q11.2	0.625	0.0241
	D-amino-acid oxidase	NM_001917	12q24	1.75	0.03
	chromosome X open reading frame 2	NM_001586	Xq28	0.762	0.0308
	transmembrane 7 superfamily member 1 (upregulated in kidney)	NM_003272	1q42-q43	0.761	0.0371
	ribonuclease, RNase A family, 4	NM_002937	14q11.1	1.279	0.0467

Genes are ranked within each category according to their significance.

**Table 2:** Summary of microarray results by gene category.

Gene Category	Genes Upregulated	Genes Downregulated
Protein Transport	6	2
Ion Transport	6	3
Proliferation	2	2
Differentiation	2	2
Anti-apoptosis/Survival	3	1
Transcription	10	4
Metabolism	5	6
Structural	5	4
Adhesion	4	1
Protein Synthesis	7	0
Immune Response & Inflammation	4	1
Signal Transduction	5	3
Cell Cycle & DNA repair	5	2
Miscellaneous	3	4

### Verification of Selected Genes by Real-time PCR

In order to corroborate the microarray gene expression results genes were selected for validation by real-time RT-PCR using the same patient samples. Miron *et al *have demonstrated that the popular strategy of selecting genes with the greatest fold-increase generally fails as a global validation strategy while a random selection of genes for validation (10–25 genes) is a more robust technique [25]. We therefore used random-stratified sampling as a gene selection method. Ten genes representing a range of p-values were randomly selected for quantitative RT-PCR analysis.

The RT-PCR expression values were calculated from a standard curve and the mean of the values calculated for the coeliac samples was divided by the mean for the control samples to give a ratio. The RT-PCR ratios were then compared to the microarray ratios (Table 3). Eight of the ten genes showed up-regulation of expression in coeliac disease with both microarray and real-time RT-PCR analysis. One gene showed up-regulation of expression in coeliac disease with microarray analysis and non-differential expression with real-time RT-PCR. One gene showed down-regulation of expression in coeliac disease with microarray analysis and up-regulation of expression with real-time RT-PCR analysis. Thus, 80% of the genes tested in this study were found to be validated, which compares well to a reported average RT-PCR confirmation rate of 70% [26].

**Table 3:** Comparison of fold-changes obtained for genes by microarray and by TaqMan RT-PCR

Gene	RT-PCR fold-change	Microarray fold-change
Peroxisome biogenesis factor 13 (PEX13)	1.62	2.28
Gap junction protein, alpha 4, (GJA4, aka connexin 37)	4.09	1.95
Syntaxin 3A (STX3A)	1.52	1.81
3-hydroxy-3-methylglutaryl-Coenzyme A synthase 1 (HMGCS1)	1.25	0.54
Sterol regulatory element binding transcription factor 1 (SREBF1)	1.26	4.53
Nudix (nucleoside diphosphate linked moiety X)-type motif 2	3.02	2.21
Small proline-rich protein 3 (SPRR3, aka Esophagin)	1.77	2.1
Sialic acid binding Ig-like lectin 6 (SIGLEC6)	3.14	1.89
Laminin 5, alpha 3 (LAMA3)	1.94	2.51
Low density lipoprotein receptor-related protein 6 (LRP6)	1.04	2.12

### Immunohistochemical Analysis

Two of the genes validated by real-time RT-PCR, were selected for immunohistochemical examination based upon their expression in the gastrointestinal tract and potential interest with respect to coeliac disease pathophysiology. Small proline-rich protein 3 (SPRR3) was selected for qualitative analysis because of its role as a structural protein and its potential to act as a substrate for tissue transglutaminase [27]. Gap junction protein alpha 4 (GJA4) was selected because of recent literature suggesting the importance of gap junctions in the spreading of immune signals between epithelial cells, including small peptides [28].

**Figure 3:**
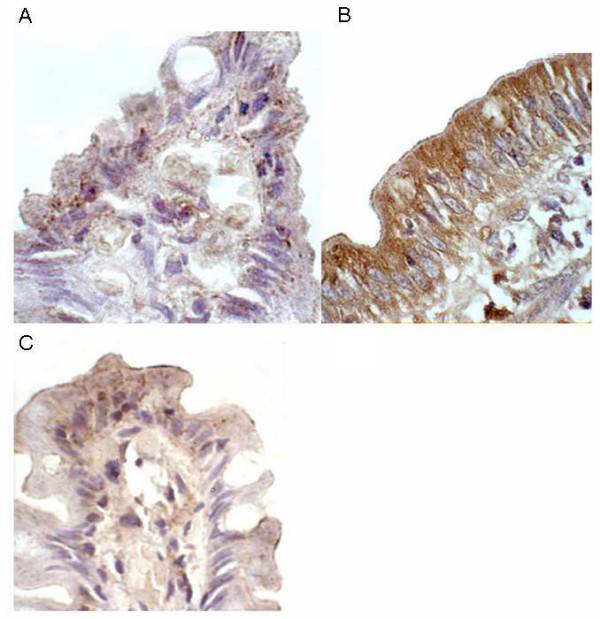
**Immunohistochemical detection of SPRR3 in duodenal tissue**. **A**: treated coeliac, showing faint staining in villous-tip epithelial cells, with occasional punctate staining of moderate intensity; **B**: untreated coeliac (same patient as A) showing intense cytoplasmic staining throughout epithelial cells with strongest staining observed on apical side of cell; and **C**: normal control showing minimal staining of epithelium with only villous-tip involved.

Protein expression of SPRR3 was examined in biopsies from five further untreated coeliac patients and in corresponding biopsies taken from these same patients at a later date while consuming a gluten-free diet (Figure 3). Histology reports showed that while consuming gluten, all patients had Marsh 3 type lesions (one Marsh 3a, 2 Marsh 3b and 2 Marsh 3c). Upon adoption of a gluten-free diet, two patients had recovered to normal duodenal histology while three patients had recovered to type 3a lesions with only mild or variable villous blunting. These patients had been following a gluten-free diet for a mean of 7 years (range 4–11 years).

In the untreated coeliac tissue the common finding was intense cytoplasmic staining of SPRR3 throughout the epithelial cell, with strongest staining observed on the apical side of the cell. Perinuclear staining was also detected, although less visible due to the intensity of the cytoplasmic staining. The staining was observed all along the epithelium with the top part of the villous showing the greatest intensity. Staining for SPRR3 was increased in the untreated coeliac disease samples when compared to the gluten-free diet samples in 4 out of 5 cases. In the fifth matched sample, staining was equivalent pre and post-treatment with the gluten-free diet. The treated coeliac tissue generally showed faint staining in the villous-tip epithelial cells, with occasional punctate staining of moderate intensity also occurring only in the villous-tip region. Biopsies from the five untreated coeliac patients who showed up-regulation of SPRR3 mRNA expression by microarray and RT-PCR analysis were also examined for protein expression of SPRR3. All five samples showed a similar intense staining picture as seen in the untreated coeliac biopsies described above. Biopsies from ten normal control patients and ten disease control patients (consisting of five with peptic duodenitis and five with Crohn's disease with duodenal involvement) showed minimal staining of the epithelium with only the villous-tip involved, in a similar pattern to that found in the treated coeliac tissue (data not shown).

**Figure 4:**
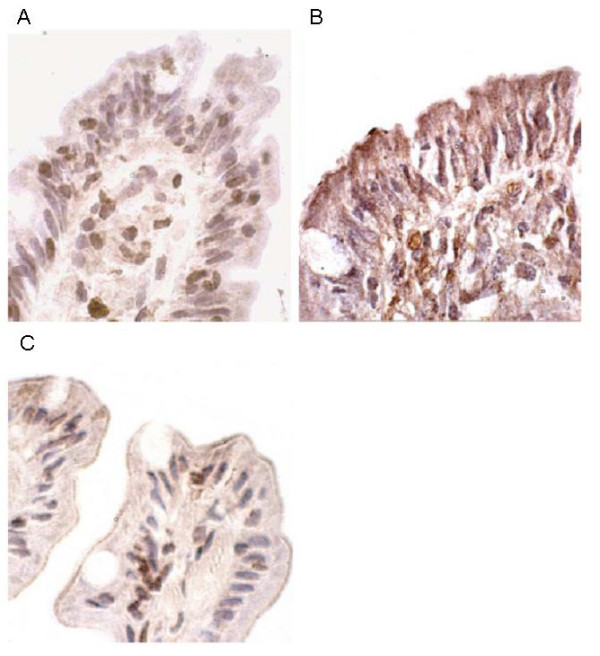
**Immunohistochemical detection of GJA4 in duodenal tissue**. **A**: treated coeliac, nuclear staining of epithelial cells apparent while brush border staining absent; **B**: untreated coeliac (same patient as A), granular staining predominantly seen in cytoplasm of epithelial cells with increasing intensity towards brush border. Perinuclear staining visible in some cells although nuclear staining generally absent. **C**: normal control, showing perinuclear staining in the epithelium, similar to treated coeliac tissue, but with less frequent nuclear staining.

Expression of GJA4 protein was compared between the matched pairs of untreated and treated coeliac biopsies (Figure 4). In the untreated epithelial tissue, granular staining was predominantly seen in the cytoplasm of the epithelial cells, with an increasing intensity towards the brush border. Perinuclear staining was visible in some cells although nuclear staining was generally absent. In contrast, in the treated biopsies, nuclear staining of epithelial cells was common while weak cytoplasmic staining was only evident in the villous tips and brush border staining was absent. The intra-epithelial lymphocytes within the treated tissue showed the most intense nuclear staining while the staining of enterocytes appeared to be due to strong perinuclear staining. In biopsies from the five untreated coeliac patients, who showed up-regulation of GJA4 mRNA expression by microarray and RT-PCR analysis, staining was similar to that seen in the untreated coeliac biopsies described above. Biopsies from control patients, both normal and diseased, showed a similar staining pattern to that observed in treated coeliac tissue although fewer cells appeared to stain positively in these control tissues (data not shown). In particular, nuclear staining was less frequent than in the treated coeliac tissue while perinuclear staining remained a common finding.

## Discussion

This is the first study to examine the gene expression profile of a highly pure population of duodenal epithelial cells in active coeliac disease. Differences in gene expression between epithelial cells of coeliac patients on a normal, gluten containing diet and non-coeliac control patients also consuming gluten were measured. Many of the genes identified in the study are known to be expressed in intestinal epithelial cells, [29-35] adding validity to the results profile described here. Of the 3,800 genes present on the array, 102 genes were found to have significantly altered expression in active coeliac disease. Of these, ten genes were selected for validation by real-time RT-PCR quantification and eight of the 10 showed up-regulation in both detection systems. Protein expression of the gene product was investigated in the case of two up-regulated genes and was increased in both.

The genes which demonstrated altered expression included those involved in cell proliferation, cell differentiation and cell death, all events which play a key role in coeliac pathology. In the villous crypt compartment, stem cells continuously proliferate to provide sufficient cells for the epithelium to renew every five days [36]. Survival of these crypt cells is key for the epithelium to maintain its self-renewing capacity. Crypt hyperplasia is a further important feature of the coeliac intestinal lesion and is said to be the first architectural change in the pathogenic process [37]. In active coeliac disease, it has previously been noted that epithelial crypt cells proliferate at an increased rate [38,39]. In this study, genes for growth arrest-specific 6 (GAS6) and bone marrow stromal cell antigen 2 (BST2) were found to be up-regulated in active coeliac disease, and both have previously been reported to induce cell proliferation [40,41]. In addition, the PRKC apoptosis WT1 regulator gene (PAWR), known to act as a negative regulator of proliferation [42], was found to be down-regulated. These results show that the transcriptional regulation of proliferation is altered in active coeliac disease and the findings are in keeping with those of Diosdado *et al *[17] who also reported altered expression of genes which lead to increased cell proliferation.

Differentiation is a key process in the intestinal epithelium, whereby immature crypt cells become specialised into mature enterocytes when they migrate up to the villous compartment. Altered expression of several genes which could affect this process was found in this study. Wnt7a was found to be up-regulated and this signalling pathway is known to have an important role in differentiation and embryogenesis [43,44]. The importance of the Wnt pathway in intestinal epithelium has been demonstrated in animal studies. In a study by Kuhnert (2004) the antagonism of Wnt signalling resulted in a marked decrease in intestinal epithelial proliferation and a degeneration of intestinal architechture [45]. While up-regulation of Wnt7A expression may provide a differentiation signal to epithelial cells, signalling via Wnt7a has also been shown to induce transcription of matrix metallproteinase 12 (MMP-12) [17], an enzyme that has been implicated in coeliac disease pathophysiology [46].

Altered expression of other genes points to a down-regulation of epithelial differentiation. For example, nuclear transcription factor Y alpha, a protein that has been shown to induce the expression of differentiation markers on CaCo-2 cells [47], was found to be down-regulated in active coeliac disease. Expression of retinol binding protein 1 (RBP1) was also found to be reduced in active coeliac disease epithelium. Retinoids play an important role in fundamental physiological processes including differentiation of epithelial tissues. RBP1 is a protein that binds metabolites of vitamin A, which has been shown to play a role in the differentiation of epithelial cells. Given that altered retinol metabolism is thought to play a part in oncogenesis and RBP1 expression is lost in epithelial cells of ovarian cancer (Cvetkovic 2003) lower levels of RBP1 may be associated with the increased chance of malignancy in coeliac disease. Overall, these results provide evidence for decreased differentiation of epithelial cells in coeliac disease.

Several of the genes found to have altered expression in this study would favour a decrease in apoptotic events. These include the up-regulation of TNFRSF18 (also known as glucocorticoid-induced TNFR-related gene) and the survival factor erythropoietin which has been shown to be involved in survival of human breast and cervix carcinoma cells [48]. Moreover, protein tyrosine kinase 2 beta (PTK2B), which enhances apoptosis [49] was found to be down-regulated in active coeliac disease. These findings concur with an earlier microarray study which found evidence of activation of the NFκB pathway [17] which can enhance cell survival by counteracting cell-death pathways [50]. Since crypt epithelial cells are in a hyper-proliferative state in coeliac disease, enhancement of cell survival by blocking apoptosis makes biological sense. Although an increase in enterocyte apoptosis has been reported in coeliac disease [18,51,52], this is not pronounced and in one study only 2.4% of cells were apoptotic [51]. Nonetheless, a certain level of apoptosis is to be expected, as terminally differentiated cells are extruded into the intestinal lumen from the villous tip. BRCA1 associated protein 1 (BAP1) was shown to have a significantly elevated expression level in this study (1.316 fold-change, p = 0.0185). This replicates a result generated by Juuti-Uusitalo *et al *(2007) [53]. The significance of this result is unclear but BAP1 has been described as a candidate tumour suppressor gene.

A number of genes involved in transport and metabolism were also found to be differentially expressed. As the epithelial layer in active coeliac disease is in disarray, the normal function of these cells is likely to become disordered. Such changes in metabolism may reflect the modified needs of rapidly renewing cells. Ion transport is known to be increased in coeliac disease [54] and an up-regulation of genes involved in ion channels was found in this study. Examples of these include genes for the potassium voltage-gated channel, Isk-related family, member 1 (KCNE1) and calcium channel, voltage-dependent, L type, alpha 1C subunit (CACNA1C). A further gene involved in regulating iron transport, HFE, which is located on chromosome 6p21 (CELIAC locus 1) was also found to be up-regulated. Interestingly, over-expression of HFE can lead to reduced iron uptake [55], another known feature of coeliac disease. Juuti-Uusitalo *et al*. [24] also reported up-regulation of genes affecting transport in coeliac patients and in particular the transcription of several ion pumps were upregulated.

Recent publications have suggested that activation of the innate immune system in the epithelium of the small intestine is a feature of coeliac disease. In this study we identified 5 genes with altered expression that are involved in the immune response. Mouse studies suggest that one of the genes, MAP/microtubule affinity-regulating kinase 2 (MARK2), plays a role in maintenance of immune system homeostasis and prevention of autoimmunity [56]. The decreased expression of MARK2 may be associated with the generation of autoimmunity in coeliac disease. Another gene product, sialyltransferase 1 (beta-galactoside alpha-2,6-sialyltransferase), appears to be involved in the sialylation of O-glycans during the process of dendritic cell maturation [57]. Increased expression of this protein may reflect the maturation of antigen presenting cells in the inflammatory lesion.

In recent studies of coeliac disease, genes involved in the intestinal barrier have been examined. One study reported an increased association with the gene for myosin 9B (MyO9B), involved in actin remodelling of epithelial cells [58]. A number of other studies, however, have not confirmed this association [59,60]. In this study, no significant alteration of expression of MyO9B was found and a fold-change of just 1.1 was noted. Some other genes, potentially involved in tight junction formation, did show an altered expression profile, although this did not reach statistical significance; these were myosin 7A (1.5 up-fold), claudin-5 (1.23 down-fold), cadherin-10 (1.39 down-fold) and actintin, alpha 1 (1.79 down-fold).

Small proline-rich protein 3 (SPRR3) was found to have increased gene and protein expression in this study. Intense cytoplasmic staining of this protein was noted in the epithelial cells of untreated coeliac patients. SPRR3, also known as esophagin, is a structural protein and a member of the cornified cell envelope precursor family. The cornified cell envelope provides a vital physical barrier in certain specialised epithelia normally subjected to mechanical trauma. The envelope is assembled by transglutaminase cross-linking of several proteins including SPRR3, which has been shown to be a substrate for transglutaminase 1, 2 and 3 [27]. Expression of SPRR3 is normally found in terminally differentiated epithelia such as the oesophagus but is up-regulated in response to epithelial injury or disease [27]. The up-regulation of SPRR3 in active coeliac disease may be a defensive response to protect the mucosa from any further damage caused by the ingestion of gluten. The increased expression of transglutaminase 2 found in the enterocytes and basement membrane in active coeliac disease [61-63] could cross-link with SPRR3 to form a cornified envelope-like barrier. While SPRR3 is clearly upregulated in enterocytes in the coeliac gut, it is not clear whether this response is gluten-specific or the result of the architechtural changes typical of coeliac disease. However, it is clear that SPRR3 protein is not expressed to the same degree in disease control samples.

Gap junction protein, alpha 4 (GJA4) was also shown to have increased gene expression and altered protein expression in patients with active coeliac disease. Granular GJA4 protein staining was found in these patients with an increase towards the brush border. GJA4, also known as connexin 37, is a member of the connexin family of gap junction structural proteins. Gap junctional intercellular communication can play various roles in terms of cell proliferation, migration and differentiation [64] and in atherosclerosis studies, connexin 37 expression has been shown to change location as the disease progresses [65]. The potential influence of gap junctions on the immune system is frequently overlooked. These channels can facilitate the transfer of small molecules like ions, metabolites and peptides up to around 16 amino acids in length [28]. Gap junctions may function as a method to spread immunological signals from, for example, viral infections from cell to cell towards an antigen presenting cell such as an interdigitating dendritic cell. The up-regulation of gap junction proteins may reflect a response to the local inflammatory mileu. Increased numbers of gap junctions could facilitate the passage of immunostimulatory gluten peptides between cells along the epithelial boundary.

## Conclusion

This study investigated gene expression in highly purified enterocytes from the duodenal biopsies of patients with untreated coeliac disease and compared the findings with age and sex-matched control subjects. By focusing on a single cell population, in contrast to analysis of whole biopsy tissue it was possible to exclude the contribution of genes expressed in a diverse range of other cell types within the coeliac lesion. Of the 102 genes found to have significantly altered expression, several code for pathological processes known to contribute to coeliac disease and other genes were identified which have not previously been associated with this disorder. Of the ten genes investigated by real-time RT-PCR, validation of altered gene expression was confirmed in 80% and in the case of two proteins, increased duodenal expression was confirmed by immunohistochemistry. The study demonstrates how the application of microarray technology to the investigation of a complex genetic disease such as coeliac disease can contribute to the elucidation of potential disease mechanisms.

## Methods

### Patients

Duodenal biopsy specimens were obtained from each patient via oesophago-gastro-duodenoscopy. Five coeliac patients had active, untreated disease and five age and sex-matched patients undergoing endoscopy for investigation of upper gastrointestinal symptoms were used as a control group. The demographic and clinical details of these patients are given in Table 4. Eight biopsy samples from each patient were used in microarray and RT-PCR experiments and a further two biopsies were processed for routine histological evaluation.

**Table 4:** Microarray Experiment Patient Clinical Details

Patients	Sex	Age	Duodenal histology	Other histology, clinical information	Antibody status	Microarray number
Coeliac						
1	Female	61	Grade 3a	none	tTG +	1
2	Female	45	Grade 3b	Barrett's oesophagus	tTG +	2
3	Female	22	Grade 1	none	tTG +	3
4	Female	31	Grade 3c	none	tTG +	4
5	Female	36	Grade 3c	mild reflux oesophagitis	tTG +	5

Control						
6	Female	58	NDM	mild reflux oesophagitis	nk	1
7	Female	51	NDM	moderate reflux oesophagitis	tTG -	2
8	Female	25	NDM	none	nk	3
9	Female	30	NDM	superficial acute and chronic gastric inflammation	nk	4
10	Female	26	NDM	mild reflux oesophagitis	tTG -	5

Histology grade: 0 = normal; 1 = raised intraepithelial lymphocytes (IELs); 2 = increase in IELs with crypt hyperplasia; 3a = increase in IELs, crypt hyperplasia, mild villous atrophy; 3b = increase in IELs, crypt hyperplasia, marked villous atrophy; 3c = total villous atrophy [37]. NDM = normal duodenal mucosa. tTG, tissue transglutaminase antibody. nk, not known.

In the immuno-histochemical studies, duodenal biopsy samples from three further groups of patients were employed. Archived tissue blocks in St. James's Hospital, Dublin were the source of these samples. The study subjects included five additional coeliac patients (3 males, 2 females, mean age 60 years): samples taken before they had commenced a gluten free diet and after dietary treatment (mean 7 years) were investigated. Two disease control patient groups were also studied: these included five patients (2 female, 3 male, mean age 51 years) with Crohn's disease involving the duodenum; and five patients (2 female, 3 male, mean age 43 years) with peptic duodenitis. Finally, a further ten patients (6 female, 4 male, mean age 59 years) undergoing endoscopy for investigation of upper gastrointestinal symptoms were also studied: this entire latter group had normal duodenal histology.

Ethical approval for this study was granted by the Joint Ethics Committee of St James's Hospital and Tallaght Hospital, Dublin, Ireland.

### Isolation of epithelial cells

Enterocyte isolation was perfomed based upon previously published methods [20]. The eight biopsies taken from each patient were transferred into RPMI culture medium containing 10% fetal calf serum and processed immediately. Biopsies were agitated in calcium and magnesium free HBSS (Gibco BRL, Scotland) containing 1 mM dithiothreitol (DTT) (Sigma, USA) and 1 mM EDTA (Sigma, USA) and incubated at 37°C for 40 minutes. The supernatant containing the epithelial cells was removed, washed twice and centrifuged at 800 rpm for 10 minutes. Cells were magnetically sorted to deplete CD3^+ ^T-cells, using MACS CD3 microbeads (Miltenyi Biotech, Germany) and an LD depletion column according to manufacturer's specifications (Miltenyi Biotech, Germany). Employing the anti-epithelial cell monoclonal antibody Ber-EP4 and the anti-T-cell monoclonal antibody CD3 (DAKO, Denmark), FACS analysis was performed on the eluted fraction in order to determine purity and values of 98–99% were repeatedly observed.

### RNA extraction

Total RNA was extracted from the epithelial cells using the NucleoSpin^® ^RNA II kit (BD Biosciences Clontech, UK) according to manufacturer's instructions with the following exceptions: vigorous vortexing of cells in the lysis buffer was performed, after which samples were frozen and then thawed before continuing with extraction; RNA was eluted from the column in 40 μl RNase-free water and the eluate reloaded onto the column twice more in order to collect the maximum yield of RNA. The RNA was concentrated further using Microcon YM-30 concentrators (Millipore, Ireland). Total RNA was quantified at 260 nm, and the 260/280 nm ratio was measured to calculate purity of RNA from contaminating protein. Agarose gel electrophoresis was also carried out to assess the quality of RNA. The same RNA samples were used for both microarray and RT-PCR analysis.

### Synthesis of fluorescent-labelled cDNA probes

Fluorescent-labelled cDNA probes were synthesised using the BD Atlas™ SMART™ Fluorescent Probe Amplification Kit (BD Biosciences Clontech, UK). Briefly, cDNA was reverse-transcribed from total RNA and purified from unincorporated nucleotides before amplification using the CyScribe GFX Purification Kit (Amersham Biosciences, UK). From each patient specimen, two probe samples were synthesised. For each sample, the optimum number of PCR cycles was determined in order to ensure the amplification process was stopped while still in the exponential phase. This was essential as over-cycled cDNA which has reached the plateau phase of amplification could result in a less representative probe when examining differential gene expression. Once complete, an aliquot of each sample was analysed on a 1.2% agarose/EtBr gel under UV light, to ensure the reactions were successful. The PCR product was purified using the CyScribe GFX Purification Kit. Absorbance was read at 260 nm to calculate quantity and the 260:280 nm ratio was measured to calculate purity. Purified PCR product was fluorescently labelled, according to manufacturer's instructions. Single-use aliquots of monoreactive Cy3-NHS ester and Cy5-NHS ester dyes (Amersham Biosciences, UK) dissolved in DMSO were used. The labelled probe was purified from unreacted dye using the CyScribe GFX Purification kit and further purified from particulate matter by filtering through a 0.22 μm spin filter. Quantity and quality of the labelled probe was determined by UV/visible spectrophotometry using a Genesys 5 spectrophotometer (Thermo Electron Corporation, US). The optimal volume of labelled probe to use in the hybridisation reaction was determined on the basis of absolute optical units (OU_λ_), using the following formula:

$${\text{V}}_{\text{opt}}(\mu \text{l})=\frac{1,000\times {\text{OU}}_{\lambda }}{{\text{A}}_{\lambda }}$$

where OU_λ _for Cy3 and Cy5 is 0.01 (determined by BD Biosciences Clontech) and A_λ _is the measured absorbance maxima for each dye; 550 nm for Cy3 and 650 nm for Cy5.

### Microarray hybridisation and scanning

The microarrays used in this study were Atlas Glass Human 3.8 I microarrays (BD Biosciences Clontech, UK). Five biological replicate experiments were conducted, each one comparing one untreated coeliac sample to one control sample. In each experiment, two technical replicates were performed. Onto one microarray, Cy3-labelled coeliac cDNA and Cy5-labelled control cDNA were co-hybridised. Onto the other microarray, Cy5-labelled coeliac cDNA and Cy3-labelled control cDNA were co-hybridised. In this manner any discrepancies in rates of incorporation of the different dyes during the labelling step are controlled for. The appropriate coeliac and control probe were combined together and hybridised onto the microarray according to manufacturer's specifications. The slides were scanned using an Affymetrix^®^428™ Array Scanner. Fluorescence was measured after excitation at 532 nm and 635 nm. Separate raw images for each dye were acquired and images were analysed using the software package ImaGene^® ^5.0 (BioDiscovery, California). Quality control measures were performed on all spots to identify empty, poor and negative spots.

### Data analysis

The raw data from image analysis was normalised using the free software ArrayNorm available at  (Graz University of Technology Bioinformatics group, Austria). All the data was subjected to a background subtraction followed by a Lowess normalisation performed separately on each block (subgrid) on each microarray. Once normalised, the technical replicates were averaged and the data saved as a text file. The normalised data was analysed using the software package GeneSpring^® ^7 (Silicon Genetics, California). A custom genome was created using the genes present on the microarrays used. The data was analysed using log of ratio and the cross gene error model was turned off. The data was filtered on confidence using the t-test with a p-value of 0.05 considered significant. Significantly differentially expressed genes were grouped into functional categories using MAPPFinder  – a program that works with GenMAPP and the annotations from the Gene Ontology (GO) Consortium [66].

### Validation of data by real time RT-PCR

Genes to be validated were selected on the basis of significance and potential interest. The genes were chosen to represent a spread of p-values up to 0.1. Assays-on-Demand™ Gene Expression products (Applied Biosystems, UK) containing forward primer, reverse primer and probe labelled with FAM dye and MGB quencher in a single tube were used. These primer/probe mixtures were pre-designed by the manufacturer and pre-optimised to ensure high amplification efficiency (proprietary sequences).

Total RNA was reverse transcribed by standard methods. cDNA samples were quantified using the Fluorescent DNA Quantitation Kit (Bio-Rad, UK) according to manufacturer's specifications, and 250 pg of cDNA sample was used per reaction. Fluorescence was measured on a Tecan GENios Fluorimeter (Tecan, UK). Quantification of gene expression was carried out in the ABI TaqMan 7000 (Applied Biosystems), using purified PCR products as standards [67]. In order to compare the expression values to those obtained from the microarray analysis, the mean expression value of the five coeliac samples was divided by the mean expression value of the five control samples to give a single ratio value.

### Immunohistochemistry

Immunohistochemical staining was performed on 3 μm thick sections, cut from formalin-fixed, paraffin-embedded biopsies, using the avidin-biotin-peroxidase complex detection method. Rabbit polyclonal anti-human SPRR3 (small proline-rich protein 3) (Apotech UK), was applied at a concentration of 1 in 1000 and rabbit polyclonal anti-mouse connexin 37 (Alpha Diagnostic, US) was used at a concentration of 1 in 150. The mouse connexin 37 immunogenic peptide has an 87% homology to human connexin 37. The selection of these two antibodies was based on their known ability to react with human tissue [68,69]. The staining procedure was carried out using the VECTASTAIN^® ^Universal Elite ABC-Peroxidase Kit (Vector Labs, USA), according to manufacturer's specifications. Slides were visualised using a Nikon eclipse TE300 microscope attached to a computer. Images were captured using Leica DC100 and Adobe Photoshop software.

## Authors' contributions

SB carried out the sample processing, RNA isolation and hybridisation, data analysis, RT-PCR, immunohistochemistry and drafted the manuscript. GB critically revised the manuscript. JK and JJ advised on study design and development. CF co-ordinated the study, arranged sample acquisition, revised and finalised the manuscript. All authors read and approved the final manuscipt.
